# Supplementary material for: IMPA1 dependent regulation of phosphatidylinositol 4,5-bisphosphate and calcium signalling by lithium
Source: Life Sci Alliance. 2023 Dec 6;7(2):e202302425. doi: 10.26508/lsa.202302425 (PMC10700560; doi:10.26508/lsa.202302425)
Supplement: Supplementary file 2 [file LSA-2023-02425_TableS2.docx]

Table S2: List of antibodies used for immunocytochemistry-

| **Primary antibody** | **Source** | **Company** | **Catalogue number** | **Dilution** |
| --- | --- | --- | --- | --- |
| SOX1 | Rabbit | Abcam | ab87775 | 1:400 |
| Nestin | Mouse | Thermo Fisher | MA1-110 | 1:100 |
| SOX2 | Mouse | Thermo Fisher | MA1-014 | 1:500 |
| Pax6 | Rabbit | Thermo Fisher | MA1-109 | 1:100 |
| GFAP | Rabbit | Abcam | ab7260 | 1:1000 |
| MAP2 | Chicken | Novus Biologicals | NB300-213 | 1:1000 |
| CTIP2 | Rat | Abcam | ab18465 | 1:800 |
| Synapsin | Rabbit | Abcam | ab64581 | 1:200 |

| **Secondary antibody** | **Company** | **Catalogue number** | **Dilution** |
| --- | --- | --- | --- |
| Alexa 488- Goat α-Rabbit IgG | Life Technology | A11034 | 1:300 |
| Alexa 568- Goat α-Chicken IgG | Life Technology | A11041 | 1:300 |
| Alexa 488-Goat α-Rat IgG | Life Technology | A11006 | 1:300 |
| Alexa 568- Goat α-Mouse IgG | Life Technology | A11004 | 1:300 |
