# Supplementary material for: IMPA1 dependent regulation of phosphatidylinositol 4,5-bisphosphate and calcium signalling by lithium
Source: Life Sci Alliance. 2023 Dec 6;7(2):e202302425. doi: 10.26508/lsa.202302425 (PMC10700560; doi:10.26508/lsa.202302425)
Supplement: Supplementary file 4 [file LSA-2023-02425_TableS4.docx]

Table S4: List of Multiple reaction monitoring (MRM) values used for detection of the lipids.

| **Q1 Mass  (Da)** | **Q3 Mass  (Da)** | **Dwell time  (msec)** | **ID** | **DP  (volts)** | **EP  (volts)** | **CE  (volts)** | **CXP  (volts)** |
| --- | --- | --- | --- | --- | --- | --- | --- |
| 1041.5 | 551.5 | 35 | 32_0 PIP2 | 60 | 11 | 37 | 15 |
| 1039.5 | 549.5 | 35 | 32_1 PIP2 | 60 | 11 | 37 | 15 |
| 1069.5 | 579.5 | 35 | 34_0 PIP2 | 60 | 11 | 37 | 15 |
| 1067.5 | 577.5 | 35 | 34_1 PIP2 | 60 | 11 | 37 | 15 |
| 1065.5 | 575.5 | 35 | 34_2 PIP2 | 60 | 11 | 37 | 15 |
| 1103.5 | 613.5 | 35 | 37_4 PIP2 | 60 | 11 | 37 | 15 |
| 995.5 | 613.5 | 35 | 37_4 PIP | 140 | 12 | 29 | 12 |
| 1097.5 | 607.5 | 35 | 36_0 PIP2 | 60 | 11 | 37 | 15 |
| 1095.5 | 605.5 | 35 | 36_1 PIP2 | 60 | 11 | 37 | 15 |
| 1093.5 | 603.5 | 35 | 36_2 PIP2 | 60 | 11 | 37 | 15 |
| 1091.5 | 601.5 | 35 | 36_3 PIP2 | 60 | 11 | 37 | 15 |
| 1089.5 | 599.5 | 35 | 36_4 PIP2 | 60 | 11 | 37 | 15 |
| 1125.5 | 635.5 | 35 | 38_0 PIP2 | 60 | 11 | 37 | 15 |
| 1123.5 | 633.5 | 35 | 38_1 PIP2 | 60 | 11 | 37 | 15 |
| 1121.5 | 631.5 | 35 | 38_2 PIP2 | 60 | 11 | 37 | 15 |
| 1119.5 | 629.5 | 35 | 38_3 PIP2 | 60 | 11 | 37 | 15 |
| 1117.5 | 627.5 | 35 | 38_4 PIP2 | 60 | 11 | 37 | 15 |
| 1115.5 | 625.5 | 35 | 38_5 PIP2 | 60 | 11 | 37 | 15 |
| 933.5 | 551.5 | 35 | 32_0 PIP | 140 | 12 | 29 | 12 |
| 931.5 | 549.5 | 35 | 32_1 PIP | 140 | 12 | 29 | 12 |
| 961.5 | 579.5 | 35 | 34_0 PIP | 140 | 12 | 29 | 12 |
| 959.5 | 577.5 | 35 | 34_1 PIP | 140 | 12 | 29 | 12 |
| 957.5 | 575.5 | 35 | 34_2 PIP | 140 | 12 | 29 | 12 |
| 989.5 | 607.5 | 35 | 36_0 PIP | 140 | 12 | 29 | 12 |
| 987.5 | 605.5 | 35 | 36_1 PIP | 140 | 12 | 29 | 12 |
| 985.5 | 603.5 | 35 | 36_2 PIP | 140 | 12 | 29 | 12 |
| 983.5 | 601.5 | 35 | 36_3 PIP | 140 | 12 | 29 | 12 |
| 981.5 | 599.5 | 35 | 36_4 PIP | 140 | 12 | 29 | 12 |
| 1017.5 | 635.5 | 35 | 38_0 PIP | 140 | 12 | 29 | 12 |
| 1015.5 | 633.5 | 35 | 38_1 PIP | 140 | 12 | 29 | 12 |
| 1013.5 | 631.5 | 35 | 38_2 PIP | 140 | 12 | 29 | 12 |
| 1011.5 | 629.5 | 35 | 38_3 PIP | 140 | 12 | 29 | 12 |
| 1009.5 | 627.5 | 35 | 38_4 PIP | 140 | 12 | 29 | 12 |
| 1007.5 | 625.5 | 35 | 38_5 PIP | 140 | 12 | 29 | 12 |
| 706.5 | 551.5 | 35 | PE 32_0 | 140 | 12 | 29 | 12 |
| 704.5 | 549.5 | 35 | PE 32_1 | 140 | 12 | 29 | 12 |
| 734.5 | 579.5 | 35 | PE 34_0 | 140 | 12 | 29 | 12 |
| 732.5 | 577.5 | 35 | PE 34_1 | 140 | 12 | 29 | 12 |
| 730.5 | 575.5 | 35 | PE 34_2 | 140 | 12 | 29 | 12 |
| 762.5 | 607.5 | 35 | PE 36_0 | 140 | 12 | 29 | 12 |
| 760.5 | 605.5 | 35 | PE 36_1 | 140 | 12 | 29 | 12 |
| 758.5 | 603.5 | 35 | PE 36_2 | 140 | 12 | 29 | 12 |
| 756.5 | 601.5 | 35 | PE 36_3 | 140 | 12 | 29 | 12 |
| 754.5 | 599.5 | 35 | PE 36_4 | 140 | 12 | 29 | 12 |
| 790.5 | 635.5 | 35 | PE 38_0 | 140 | 12 | 29 | 12 |
| 788.5 | 633.5 | 35 | PE 38_1 | 140 | 12 | 29 | 12 |
| 786.5 | 631.5 | 35 | PE 38_2 | 140 | 12 | 29 | 12 |
| 784.5 | 629.5 | 35 | PE 38_3 | 140 | 12 | 29 | 12 |
| 782.5 | 627.5 | 35 | PE 38_4 | 140 | 12 | 29 | 12 |
| 780.5 | 625.5 | 35 | PE 38_5 | 140 | 12 | 29 | 12 |
| 690.5 | 535.5 | 65 | PE(31:1) | 140 | 12 | 29 | 12 |
